# The Native Fruit *Geoffroea decorticans* from Arid Northern Chile: Phenolic Composition, Antioxidant Activities and In Vitro Inhibition of Pro-Inflammatory and Metabolic Syndrome-Associated Enzymes

**DOI:** 10.3390/molecules22091565

**Published:** 2017-09-18

**Authors:** Felipe Jiménez-Aspee, Cristina Theoduloz, Maria del Pilar C. Soriano, Maider Ugalde-Arbizu, Maria Rosa Alberto, Iris Catiana Zampini, Maria Inés Isla, Mario J. Simirgiotis, Guillermo Schmeda-Hirschmann

**Affiliations:** 1 Departamento de Ciencias Básicas Biomédicas, Facultad de Ciencias de la Salud, Universidad de Talca, Talca 3460000, Chile; 2 Laboratorio de Cultivo Celular, Facultad de Ciencias de la Salud, Universidad de Talca, Talca 3460000, Chile; 3 Laboratorio de Química de Productos Naturales, Instituto de Química de Recursos Naturales, Universidad de Talca, Talca 3460000, Chile; mcaramantin@utalca.cl (M.d.P.C.S.); ugamai@hotmail.com (M.U.-A.); schmeda@utalca.cl (G.S.-H.); 4 Laboratorio de Investigación de Productos Naturales (LIPRON), Instituto de Química del NOA (INQUINOA, CONICET), Universidad Nacional de Tucumán, San Miguel de Tucumán, Tucumán 4000, Argentina; mralberto@fbqf.unt.edu.ar (M.R.A.); zampini@csnat.unt.edu.ar (I.C.Z.); misla@tucbbs.com.ar (M.I.I.); 5 Laboratorio de Productos Naturales, Instituto de Farmacia, Facultad de Ciencias, Universidad Austral de Chile, Valdivia 5090000, Chile; mario.simirgiotis@uach.cl

**Keywords:** *Geoffroea decorticans*, antioxidant capacity, anti-inflammatory activity, phenolic composition, metabolic syndrome-associated enzymes, HPLC-DAD-ESI-MS

## Abstract

The native tree *Geoffroea decorticans* (chañar) grows in the arid lands of northern Chile. It has been used as a food plant since prehistoric times. Phenolic-enriched extracts (PEEs) of Chilean chañar fruits were assessed for their chemical composition, antioxidant properties and inhibition of pro-inflammatory and metabolic syndrome-associated enzymes. Phenolic profiles were determined by HPLC-DAD-MS/MS. The PEEs of *G. decorticans* showed a strong effect towards the enzymes COX-1/COX-2, with inhibition percentages ranging from inactive to 92.1% and inactive to 76.0% at 50 µg PEE/mL, respectively. The IC_50_ values of the PEEs towards lipoxygenase and phospholipase A2 inhibitory activity were between 43.6–96.8 and 98.9–156.0 μg PEE/mL, respectively. Samples inhibited α-glucosidase (IC_50_ 0.8–7.3 μg PEE/mL) and lipase (9.9 to >100 μg PEE/mL). However, samples did not inhibit α-amylase. The HPLC-DAD-MS analysis of the PEEs allowed the tentative identification of 53 compounds, mainly flavonol glycosides and procyanidins. The procyanidin content of the Chilean *G. decorticans* pulp was positively correlated with the antioxidant activity and the inhibition of the enzyme α-glucosidase. These results indicate that the Chilean chañar fruit contains bioactive polyphenols with functional properties.

## 1. Introduction

**Figure 1:**
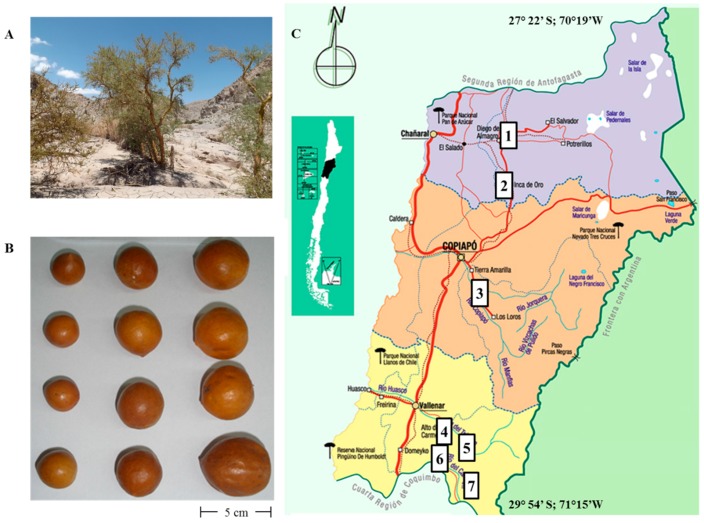
(**A**) Chañar tree (*Geoffroea decorticans*) growing in the Región de Copiapó; Chile (**B**) Different fruits showing variation in size (**C**) Map of Chile showing the Región de Atacama and the collection places of chañar fruits. Provincia de Chañaral: (1) Diego de Almagro and (2) Inca de Oro; Provincia de Copiapó: (3) Copiapó (ripe and turning fruits); Provincia de Huasco: (4) Alto del Carmen; (5) El Transito; (6) Pinte and (7) Conay.

Due to global climate change, food plants from arid zones are becoming increasingly relevant as food sources worldwide. Plants adapted to harsh environments with reduced water requirements were selected by the Native South Americans as valuable plants in the dry areas of the continent. Some of them can be found semi-domesticated in or nearby the former Amerindian settlements, who helped in their distribution through gathering and trading activities. Among them, the tree *Geoffroea decorticans* (Gill. ex Hook. et Arn.) Burkart (Fabaceae), popularly known as chañar, is a characteristic species of South America, including the arid northern Chile [1,2]. The fruits of *G. decorticans* are brown spherical drupes with a sweet pulp and pleasant taste (Figure 1A,B). In Chile, it can be found in the northern part of the country, between the Provincias de Arica and Choapa, growing on sandy plains, oases and streams from sea level up to 2300 m.a.s.l. [2]. The fruits can be eaten raw, boiled or in preserves. The husk, leaves and flowers are used in traditional medicine, boiled and generally mixed with sugar or honey for the treatment of respiratory and digestive illnesses [2].

The antioxidant activity, total phenolic and flavonoid content, acute toxicity in rodents, as well as the antinociceptive effect of Argentinean *G. decorticans* fruits has been reported [3]. The polyphenol-enriched fraction of the Argentinean fruit flour showed an inhibitory effect on key enzymes involved in metabolic syndrome, such as α-glucosidase, α-amylase, lipase and HMG-CoA reductase, and also against the pro-inflammatory enzymes lipoxygenase, cyclooxygenase-1 and -2, and phospholipase A2 [4]. The proximate composition, fatty acids and sterol constituents of the seeds of Argentinean *G. decorticans* was reported and the authors concluded that this species is a good oil source candidate [5]. Isoflavanones, including prenyl derivatives, were isolated from the bark [5]. All the studies on *G. decorticans* fruits have been carried out with single collections of Argentinean samples [6]. However, to the best of our knowledge, no information was found regarding bioactivity, nutraceutical potential and chemical composition of the *G. decorticans* fruits from Chile. The distribution of this tree on both sides of the Andes Mountains may have led to speciation or to the development of chemotypes since the Chilean populations are more isolated, growing in the longitudinal valleys from the Andes to the Pacific Ocean. Thus, the aim of this study was to describe the phenolic composition of *G. decorticans* fruits collected in northern Chile, to assess the antioxidant capacity, inhibitory potential of the polyphenol enriched-extracts (PEEs) against pro-inflammatory and metabolic syndrome associated-enzymes, and to compare our results with the Argentinean populations of the tree.

## 2. Results and Discussion

### 2.1. General Analysis of the Fruits

Nine *G. decorticans* samples were collected from seven locations of the Región de Atacama, northern Chile. The percent of edible pulp in the fruit, yields of MeOH extract and PEE of *G. decorticans* samples are summarized in Table 1. The pulp of the fruit represents 58.3–78.1% of the fruit weight, with a lower value for Alto del Carmen and a higher proportion for the Diego de Almagro samples. From the pulp, the methanol extraction yielded from 29.1–69.2% *w*/*w* of solubles. After adsorption in Amberlite XAD7, most of the MeOH extract constituents were not retained in the resin, as can be observed in the PEE yields, ranging from 0.7 to 4.0% *w*/*w* of the fresh fruit weight. The highest PEE yield was found in the sample from Alto del Carmen (Table 1).

### 2.2. Total Phenolic (TP), Total Flavonoid (TF) and Total Proanthocyanidin (TPAC) Content

The TP content of the PEEs of *G. decorticans* fruits was variable, ranging from 196.2 to 639.2 g GAE/kg PEE, with the highest value found in the sample from Inca de Oro, and the lowest value in the sample from Conay. For the TF content, the values ranged between 37.8–260.5 g CE/kg PEE. The highest values were found in the samples from the Provincia de Chañaral, while the lowest values were from the Provincia de Copiapó. The TPAC content ranged between 11.7–123.4 g CE/kg PEE, including one sample below the detection limit (Conay). The highest value was found in the sample from Alto del Carmen. The results are summarized in Table 1.

### 2.3. Antioxidant Activity

Several chemical-based methodologies have been developed to determine the antioxidant capacity of fruits and food plants. These assays are based on different strategies providing complementary information about the interaction between radicals and samples. In the present work, we evaluated the antioxidant activity of the samples using four different assays, based on three different chemical mechanisms: the scavenging of the free radicals DPPH, the capacity to reduce ferric and cupric ions, and the scavenging of superoxide anion generated by an enzyme reaction (Table 1).

In the FRAP assay, the samples presented values between 0.9–3.1 mmol TE/g PEE. The most active samples were from Inca de Oro (3.1 ± 0.1 mmol TE/g PEE), followed by the samples from Alto del Carmen (3.0 ± 0.2 mmol TE/g PEE) and both samples (turning and ripe) from Copiapó (2.9 ± 0.1 and 2.8 ± 0.2 mmol TE/g PEE, respectively). In the CUPRAC assay the same trend was observed, with the highest values in the turning samples from Copiapó (7.4 ± 0.1 mmol TE/g PEE), followed by the sample from Inca de Oro (6.7 ± 0.3 mmol TE/g PEE) and Alto del Carmen (6.7 ± 0.1 mmol TE/g PEE). The less active samples in all the antioxidant assays were from Conay and El Transito II. Compared with other native fruits from the arid northern Chile, the *G. decorticans* PEEs are more active than *Eulychnia acida* [7] or *Prosopis* species [8]. The results are summarized in Table 1.

In the DPPH discoloration assay, *G. decorticans* PEEs presented SC_50_ values between 3.9–24.3 μg PEE/mL. The highest activity was found in the samples from the Provincia de Copiapó, while the lowest activity was the sample from Conay. The DPPH scavenging activity of Argentinean *G. decorticans* flour was reported [9], but the values reported were much higher than the Chilean samples, ranging from 18–310 μg GAE/mL, indicating a lower antioxidant capacity.

The superoxide anion generated by xanthine oxidase can be scavenged by polyphenols. In this assay, the flavonol catechin was used as a positive control, showing a SC_50_ of 8.7 ± 0.1 μg/mL. All *G. decorticans* fruit PEEs were able to scavenge the superoxide anion, with SC_50_ values between 14.5–44.2 μg/mL, with the exception of the sample from El Transito II that only scavenged 45.1% the radical at 50 μg/mL. The most active samples were from Alto del Carmen (14.5 μg/mL), Copiapó (turning; 15.2 μg/mL) and Inca de Oro (18.1 μg/mL), all being more active than other Fabaceae plants [10]. The results are summarized in Table 1.

### 2.4. Inhibition of Pro-Inflammatory Enzymes

Cytokines, leukotrienes and prostaglandins play important roles in the inflammatory response, and along with oxidative stress, is considered a key factor in the development of several chronic diseases [11]. Targeting oxidative stress-inflammatory cytokine signalling can be considered as a strategy to prevent and improve therapeutic options in certain patients. The *G. decorticans* PEEs were assessed for its capacity to inhibit the pro-inflammatory enzymes LOX, COX-1/COX-2 and sPLA_2_. These enzymes participate in the arachidonate metabolism, being responsible for the biosynthesis of inflammatory lipid mediators such as prostaglandins, thromboxanes, leukotrienes and hydroxyeicosatetraenoic acids [12].

Food polyphenols with LOX inhibitory activity have been proposed as an alternative to treat some inflammatory diseases [13]. The IC_50_ of the *G. decorticans* PEEs against LOX was in the range 43.6–96.8 μg/mL (Table 2). The highest inhibitory activity was found for Alto del Carmen, while the lowest activity was found in the sample of El Transito II. The IC_50_ of a single Argentinean sample was found to be 48 μg GAE/mL [4].

The COX-1 enzyme is constitutively expressed and plays important roles in the protection of gastric mucosa and normal platelet function. A strong inhibition of COX-1 at 50 µg/mL was observed for the ripe fruits from Copiapó (92.1%), Pinte (87.3%), and Conay (80.8%), followed by the turning fruits from Copiapó (74.8%) and El Transito II (70.1%) (Table 2).

The anti-inflammatory, analgesic and antipyretic effect of non-steroidal anti-inflammatory drugs (NSAIDs) are accomplished by the inhibition of COX-2 [12]. At 50 μg/mL, a clear differentiation in the enzyme inhibition of the ripe fruits compared with turning fruits was observed for the Copiapó samples (12.9% and 55.5% inhibition, respectively). When comparing among the ripe fruits, the best activity was found for the Conay sample, with a inhibition of 76% at 50 μg/mL. The collections from the ripe fruits of Copiapó and El Transito I inhibited the enzyme by about 50% at 50 μg/mL (Table 2).

The sPLA_2_ enzymes are responsible for the hydrolysis of cell membrane phospholipids to release arachidonic acid, the precursor of the pro-inflammatory eicosanoids [12]. All PEEs of *G. decorticans* fruits were active against the sPLA_2_ enzyme (Table 2). The most active samples were from Diego de Almagro (IC_50_ 98.9 μg/mL), Inca de Oro (IC_50_ 142.9 μg/mL) and Alto del Carmen (IC_50_ 156.0 μg/mL). At 200 μg/mL, the least active samples inhibited the enzyme by 34.6–42.8%. The reference compound, ursolic acid (50 μg/mL) only inhibited sPLA2 by 26.7%. Costamagna et al. reported an IC_50_ value of 225 μg GAE/mL for an Argentinean *G. decorticans* flour [4].

From all the samples, the most active regarding LOX, COX-1/COX-2 and sPLA_2_ was the Diego de Almagro sample. The sample from Alto del Carmen was active against LOX, COX-2 and sPLA_2_, but inactive towards COX-1. These results are interesting considering that the side effects of NSAIDs are mostly derived from the inhibition of COX-1 [14]. This sample also showed the highest antioxidant activity (Table 2). New collections of this sample are needed in order to confirm this selective and potent effect.

### 2.5. Inhibition of Metabolic Syndrome Associated-Enzymes

Naturally occurring inhibitors of the enzyme α-glucosidase and α-amylase are of interest to control post-prandial hyperglycaemia [15]. The increasing prevalence of diabetes worldwide and the search for alternative methods to control this disease encourages further work on α-glucosidase and α-amylase inhibitors from food and medicinal plants. All *G. decorticans* PEEs were able to inhibit the activity of α-glucosidase with IC_50_ values ranging from 0.7–7.3 μg/mL (Table 2). The most active samples were from Alto del Carmen (0.7 μg/mL) and Copiapó (ripe, 0.8 μg/mL; turning, 2.1 μg/mL), and were more active than acarbose (IC_50_ 120.9 μg/mL). Interestingly, all the *G. decorticans* PEEs were unable to inhibit the α-amylase enzyme at the highest concentration assayed (100 μg/mL, data not shown), while the positive control acarbose presented an IC_50_ of 28.5 μg/mL. These results are similar to those reported by Costamagna et al. [4], where a single sample of *G. decorticans* fruit extract inhibited more selectively α-glucosidase (IC_50_ 0.68 μg/mL) than α-amylase (IC_50_ 25.0 μg/mL). As for Costamagna et al. our results show that the polyphenols from *G. decorticans* selectively inhibit α-glucosidase, which may reduce the incidence of undesired side effects of acarbose in the gastrointestinal tract [4]. Compared with other fruits, *G. decorticans* samples showed higher activity against α-glucosidase. For example, the α-glucosidase inhibitory effect of wine grape pomace extracts has been reported [16]. The pomace extracts were tested at 500 µg/mL and compared with acarbose at 50 µg/mL. Five out of the six grape pomace samples inhibited the enzyme in the range of 72–95%, indicating the presence of glucosidase inhibitors. However, the phenolics used as standards for the characterization in the samples (15 phenolic antioxidant standards) did not present significant inhibition of the enzyme, suggesting the presence of unidentified α-glucosidase inhibitors or a possible synergistic effect [16]. The effect of A-type procyanidins from *Litchi chinensis* and B-type procyanidins from *Nelumbo nucifera* on the inhibition of α-glucosidase in mice was reported [17]. The study showed that A-type oligomeric procyanidins are more effective than B-type procyanidins to control hyperglycaemia in diabetic mice. The antioxidant, α-glucosidase and lipase inhibitory effect of Canadian lentil cultivars was evaluated [18]. The authors showed that the effect on α-glucosidase of the de-sugared extract was in the high range of 23.1–41.2 mg/mL. Cocoa brew inhibited α-glucosidase with a high IC_50_ of 7.87 mg/mL [19], all of this examples being much less effective than *G. decorticans* polyphenols. 

Obesity is a primary preventable risk factor in the development of metabolic syndrome [20]. Natural sources from plants to fight obesity and overweight are currently being explored [21]. All the studied *G. decorticans* PEEs were able to inhibit porcine pancreatic lipase at 100 μg/mL. The most active samples were from Inca de Oro, Alto de Carmen and Copiapó (turning) with IC_50_ values of 9.9, 14.2 and 66.0 μg/mL, respectively. The other studied samples showed less inhibitory activities within the range of 0.0–34.9% (Table 2). Under our experimental conditions, the reference compound orlistat showed an IC_50_ value of 0.04 µg/mL. Costamagna et al. reported for a single collection of Argentinean *G. decorticans* an IC_50_ of 4 μg GAE/mL towards pancreatic lipase [4]. The inhibitory activity of Canadian lentils towards pancreatic lipase was also assessed, and the IC_50_ value of the different extracts ranged from 6.26 to 9.26 mg/mL [18]. In a study of selected commercial standards of polyphenols, the authors compared the inhibitory activity of the compounds towards porcine pancreas lipase. The best enzyme inhibition was observed for the compounds containing a galloyl moiety, with gallic acid, epigallocatechin and epigallocatechin gallate presenting IC_50_ values of 387.2, 237.3 and 391.2 µM, respectively. According to the kinetic analysis, the activity seems to be competitive [22]. The synergistic effect of the flavone apigenin, the flavonol quercetin, the flavanone naringenin-7-*O*-glycoside and the anthraquinone emodin on 3T3-L1 preadipocyte differentiation and inhibition of pancreatic lipase has been reported [23]. The study showed that natural products in combinations may increase their relative effectiveness on the biological target and have potential in the prevention and treatment of obesity.

### 2.6. Tentative Identification of Phenolic Compounds in G. decorticans Fruits by HPLC-DAD and HPLC-ESI-MS/MS

**Figure 2:**
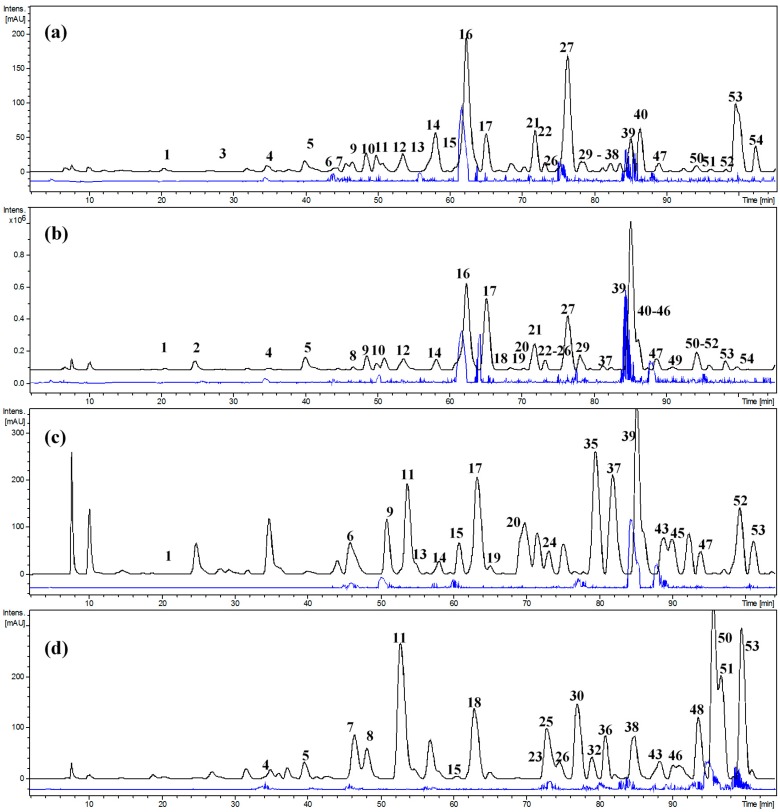
HPLC profile (280 nm, black) and total ion chromatogram (blue) of the polyphenolic-enriched extracts of *G. decorticans* frutis from: (**a**) Alto del Carmen; (**b**) Copiapó (ripe); (**c**) Sephadex fractions 20–25; (**d**) Sephadex fractions 28–30.

In this study, 53 compounds were tentatively identified in the PEEs of *G. decorticans* fruits and in the Sephadex LH-20 fractions using photodiode array detection (DAD) and negative electrospray ionization mass spectrometry in full scan mode and tandem MS/MS fragmentations (Table 3). The structures of the compounds were proposed based on UV absorption and MS fragmentation patterns. In this work using tandem MS experiments, the loss of 162 Daltons is indicative of a hexose (glucose or galactose are the most common hexoses found in flavonoids), the loss of 146 Daltons is indicative of rhamnose, the loss of 176 Daltons is indicative of glucuronic acid, the loss of 132 Daltons is indicative of a pentose (the most common pentoses found in natural products are xylose or arabinose), and the loss of 308 Daltons suggests the disaccharides rutinose (α-l-rhamnopyranosyl-(1→6)-β-d-glucopyranose) or neohesperidose (α-l-rhamnopyranosyl-(1→2)-β-d-glucopyranose) linked thorough an *O*-glycosidic bound [24]. The identification of all the detected and tentatively characterized compounds present in *G. decorticans* fruit extracts is explained below. The total ion chromatogram (TIC) and UV profile (280 nm) of *G. decorticans* PEEs from Alto del Carmen, Copiapó (ripe) and two representative Sephadex fractions from Diego de Almagro are presented in Figure 2.

#### 2.6.1. Flavonoid Derivatives

Several flavonoids, including flavonols, flavones, flavanones and flavan-3-ols, were identified in the samples submitted to HPLC-MS/MS analysis. Costamagna et al. described the presence of flavonoids in *G. decorticans* fruits and flour [4]. The compounds included the aglycones, monoglycosides and diglycosides of quercetin, kaempferol, isorhamnetin, naringenin, luteolin and eriodictyol, as follows.

Ten kaempferol derivatives were identified by the main fragment ion at *m*/*z* 285. Peak **51** was identified as kaempferol hexoside by the neutral loss of 162 Da; while peaks **34** and **36** showed the consecutive loss of two hexoses and were assigned as kaempferol dihexosides. Peaks **38** and **44** showed a [M − H]^−^ base peak at *m*/*z* 579, with the consecutive neutral loss of 132 and 162 Da, being assigned as kaempferol pentoside hexosides. Triglycosylated kaempferol derivatives were identified in peaks **22** and **27**. Peak **22** showed a [M − H]^−^ base peak at *m*/*z* 741, with the consecutive loss of 162, 132 and 162 Da, and was tentatively identified as kaempferol dihexoside pentoside, while peak **27** showed a [M − H]^−^ base peak at *m*/*z* 755, with the consecutive loss of 162 and 308 Da, being identified as kaempferol hexoside rutinoside. The last kaempferol derivative (peak **52**) showed the neutral loss of 176 Da, and was tentatively identified as kaempferol glucuronide. Peak **35** showed a [M − H]^−^ base peak at *m*/*z* 623, with the consecutive loss of 162 and 176 Da, being identified as kaempferol hexoside glucuronide. Peak **11** showed a base peak at *m*/*z* 615 leading to a MS^2^ at *m*/*z* 285, and was assigned as an unknown kaempferol derivative.

Eight quercetin glycosides were identified at [M − H]^−^ *m*/*z* 771, 625, 609, 595, 477, and 463 with main fragment ion at *m*/*z* 301. Peak **41** was identified as quercetin hexoside by the loss of 162 Da. Peak **43** showed the neutral loss of 176 Da and was tentatively identified as quercetin glucuronide. The dihexoside of quercetin were identified by the consecutive loss of two hexoses units (peak **23**), while a pentoside hexoside derivative (peak **32**) was also identified by the neutral loss of 132 and 162 Da. The main peak identified in the total ion chromatogram (peak **39**) showed a parent ion of *m*/*z* 609, with MS^2^ base peak at *m*/*z* 463 and 301, being identified as quercetin rutinoside. The identity of this compound was confirmed with the co-injection of a rutin standard (data not shown). The triglycosides of quercetin were identified by the loss of two hexoses and one rhamnose (peaks **24** and **33**).

Six isorhamnetin derivatives were identified by the base peak at *m*/*z* 315 Da. Some diglycosides were identified by the consecutive loss of 132 and 162 Da (isorhamnetin pentoside hexoside, peaks **42**, **46** and **49**). In addition, isorhamnetin rutinoside (peak **50**) was identified by the base peak at *m*/*z* 623 and the neutral loss of 308 Da. The triglycosides of isorhamnetin were also identified, including peak **45** that showed the neutral loss of 132 and 308 Da, tentatively identified as isorhamnetin pentoside rutinoside, and peak **47**, which showed the neutral loss of two 162 and 146, and was assigned as isorhamnetin dihexoside rhamnoside. 

Some less abundant flavonoids were also identified. A luteolin triglycoside (peak **40**) was identified by the base peak at *m*/*z* 285, with MS^3^ fragmentation to 241 and 175, characteristic of luteolin aglycone [25]. A myricetin hexoside was tentatively identified by its base peak at *m*/*z* 317 (peak **25**), while two naringenin hexosides (peaks **30** and **37**) and a diglycoside (peak **28**) were identified at a base peak of *m*/*z* 271. Two taxifolin hexosides (peaks **7** and **14**) were identified by their base peak at *m*/*z* 303. Additionally, two eriodictyol monoglycosides (peaks **3** and **15**) were identified by their MS peak at *m*/*z* 287 with MS^3^ fragmentation to *m*/*z* 151 Da, as reported [4].

#### 2.6.2. Flavan-3-ol Monomers and Polymers

Peaks **1** and **2** showed a base peak at *m*/*z* 301 (UV max 280 nm), with the neutral loss of 162 and 132 Da, respectively being assigned as ellagic acid hexoside and pentoside, respectively. Peak **31** also showed a base peak at *m*/*z* 301, and was identified as an ellagic acid derivative. Peaks **4**, **5** and **8** were identified as flavan-3-ol derivatives (UV max 280 nm), with pseudomolecular ions at *m*/*z* 451 and fragment MS^2^ ions at *m*/*z* 289, by the loss of a hexose moiety (162 Da), and were identified as (epi)-catechin hexoside [26]. The MS of peaks **6**, **12** and **26** and **29**, with [M − H]^−^ at *m*/*z* 577 and MS/MS fragmentation of 289 is compatible with (epi) catechin- (epi) catechin dimer, tentatively identified as a B-type procyanidin according to the fragmentation pattern [27]. Compounds **13**, **16** and **17** presented a [M − H]^−^ ion at *m*/*z* 863 with fragmentations to *m*/*z* 711, 695, 573 and 451. This fragmentation pattern is compatible with A-type procyanidin trimer with two A-type linkages [28]. The MS of compound **21** shows the [M − H]^−^ base peak at *m*/*z* 939 with the loss of [M-2CO-H_2_O, 74 Da], and the consecutive fragmentation pattern of a procyanidin trimer. Hence, the compound **21** was assigned as a procyanidin trimer derivative. Compound **19** showed a base *m*/*z* peak at 1153, with three consecutive loss of 289 Da, and was assigned as a B-type procyanidin tetramer [27]. Condensed tannins are the main polyphenolic components in a variety of plant-derived foods, including grains, berries, and nuts. They were previously described by Costamagna et al. as the main phenolics in the flour of Argentinean *G. decorticans* quantified by colorimetric means [16]. Similarly, the content of TPAC was higher than the content of flavonoids in our sample (Table 1). These compounds were not described in the HPLC-MS analysis of Argentinean *G. decorticans* flour [4]. To the best of our knowledge, this is the first attempt to describe proanthocyanidins in *G. decorticans* fruits. A broader analysis that includes the identification of mean degree of polymerization by means of thiolytical depolymerization will be carried out in future studies.

#### 2.6.3. Phenolic Acids

Peaks **9** and **10** were characterized as vanillic acid mono- and diglycosides, respectively, according to their fragmentation patterns. Peak **9** shows a base peak at *m*/*z* 329, with the neutral loss of a hexose from the mother ion, leading to vanillic acid. The compound was identified as a vanillic acid hexoside. On the other hand, peak **10** shows the consecutive loss of a hexose (−162 Da) and a pentose (−132 Da) leading to the base peak at *m*/*z* 167. The compound was identified as a vanillic acid hexoside pentoside. The compounds have been previously identified in Argentinean *G. decorticans* fruits [4]. The MS of compound **18** shows a parent ion of *m*/*z* 337, with MS^2^ base peak at *m*/*z* 191. The hierarchical scheme for the identification of phenolic acids allowed us to tentatively identify this compound as 5-*p*-coumaroylquinic acid [29]. Following the same scheme, we were able to differentiate the identity of peaks **48** and **53** that showed the same base peak at *m*/*z* 515. The fragmentation pattern of compound **48** lead to MS^2^ peaks at *m*/*z* 353, 191 and 135, compatible with 3,5-dicaffeoylquinic acid [29]. On the other hand, compound **53** showed MS^2^ peaks at *m*/*z* 353 and 173, and was tentatively identified as 4,5-dicaffeoylquinic acid [29].

### 2.7. Effect of Fractionation by Sephadex LH-20 on DPPH and α-Glucosidase Activity 

Sephadex LH-20 gel permeation has been used to fractionate phenolic compounds occurring in complex mixtures. A trend observed in the HPLC-DAD analysis after the fractionation was the simplification of profiles (data not shown). In the DPPH discoloration assay, the SC_50_ of the crude PEE from Diego de Almagro (12.1 µg PEE/mL) was higher than all the single fractions pools, suggesting a possible synergy of the different constituents in the fractions (Table 4).

A continuous increase in the antioxidant activity was observed starting from fractions 20–25, reaching the best effect in fractions 43–44 with an SC_50_ of 24.1 µg/mL. Hereinafter, the antioxidant capacity started to decline. On the other hand, the activity of the Diego de Almagro PEE towards α-glucosidase showed a strong inhibition with an IC_50_ of 4.7 µg/mL. Separation of the PEE by Sephadex LH-20 led to fraction pools with higher inhibitory effect, ranging from 0.4 to 8.0 µg/mL (Table 4). The highest activity was found between fractions 20–25 to 47–48 with IC_50_ values ranging from 0.4 to 0.9 µg/mL. The inhibitory activity of a grape seed procyanidin extract against α-glucosidase has been associated with the presence of polymeric structures [30], which are needed for the modulatory effect on glucose and lipid metabolism. The authors separated and tested the chromatographic fractions of the grape seed procyanidins extract, leading to more active fractions [30], similar to what we observed in *G. decorticans*. The HPLC-MS analysis of *G. decorticans* fractions 20–25 and 28–30 showed B-type procyanidin dimer, procyanidin A trimer, kaempferol and isorhamnetin glycosides as well as quercetin glucuronide for fraction pool 20–25 and catechin hexosides, procyanidin B dimer, glycosides of kaempferol and isorhamnetin, quercetin glucuronide and dicaffeoylquinic acids for the fraction pool 28–30, as described in the previous section. The fractions 1–9 and 10–14 were the less active in terms of bioactivity. The SC_50_ and IC_50_ values for the DPPH scavenging and α-glucosidase inhibition assays, respectively, were even higher than those obtained for the PEE. Taking these results into consideration both fractions were not further analyzed.

### 2.8. Cytotoxicity Assay

Phenolic-enriched extracts of chañar pulp were devoid of toxicity towards human MRC-5 lung fibroblast, with IC_50_ values >500 µg/mL. The results indicate that the PEEs did not interfere with basic cell functions nor affect cell viability, suggesting that the consumption of this fruit might be considered as safe. Previous studies have shown that *G. decorticans* consumption in rats was regarded as safe [3]. 

### 2.9. Statistical Analyses

The Tukey’s test showed significant differences among many of the samples in all the assays carried out. Interestingly, the turning and ripe samples of *G. decorticans* collected in Copiapó did not present significant differences in the TP and TPAC content, FRAP and DPPH antioxidant assays, and the LOX inhibitory activity assay (Table 1 and Table 2). On the other hand, the two collections of El Transito showed statistical differences in most of the assays carried out.

A Pearson correlation was used to evaluate the relationship between the individual parameters and to establish their relative importance in determining the bioactivity of the *G. decorticans* fruit extracts. The total phenolic, total flavonoid and total proanthocyanidin content were established as the independent variables and the quantitative parameters of the bioactivity, in other words, antioxidant activity, inhibition of lipoxygenase and inhibition of α-glucosidase are the dependent variables. Strong and significant correlations were found between the total phenolic content and the FRAP (*r* = 0.874, *p* < 0.01), CUPRAC (*r* = 0.773, *p* < 0.05) and the scavenging of superoxide anion (*r* = −0.821, *p* < 0.05). The total flavonoid content did not correlate with any of the bioactivity assays performed. However, the total proanthocyanidin content showed remarkable correlations (*p* < 0.01) with all the bioactivity assays, with the exception of the inhibition of LOX (*p* > 0.05). Condensed tannins isolated from several fruits have been previously correlated with the inhibitory effect against α-glucosidase [31], and the antioxidant activity [32]. A dose-response correlation with the antioxidant capacity and the tannin content of decoctions and alcoholic beverages of *G. decorticans* was reported [9]. Our observations may suggest that the presence of condensed tannins in *G. decorticans* fruits may be mainly responsible for the bioactivities assayed; however, a quantification of individual monomers and polymers should be done in order to establish a more precise correlation.

## 3. Materials and Methods

### 3.1. Chemicals

The following reagents were purchased from Sigma-Aldrich (St. Louis, MO, USA): AlCl_3_, amberlite XAD7 HP, α-amylase from porcine pancreas (A3176; EC 3.2.1.1), α-glucosidase from *Saccharomyces cerevisiae* (G5003; EC 3.2.1.20), DPPH (2,2-diphenyl-1-picrylhydrazyl radical), DMAC (4-[dimethylamino]cinnamaldehyde), 3,5-dinitrosalicylic acid, catechin, caffeic acid, CuCl_2_, hypoxanthine, indomethacin, linoleic acid (L-5900), lipase from porcine pancreas type II (L-3126; EC 3.1.1.3), Na_2_CO_3_, nimesulide, 4-nitrophenyl-α-d-glucopyranoside, nitro blue tetrazolium salt, *p*-nitrophenyl palmitate, sodium acetate, soy lipooxygenase-15 (L7395; EC 1.13.11.12), starch, 2,4,6-tri(2-pyridyl)1,3,5-triazine (TPTZ), triton X-100, tris, and ursolic acid. From Merck (Darmstadt, Germany): Trolox (6-hydroxy-2,5,7,8-tetramethylchroman-2-carboxylic acid), FeCl_3_∙6H_2_O, neocuproin, potassium sodium tartrate and HPLC-grade methanol. Ammonium acetate was from JT Baker (Xalostoc, Mexico). The sodium and potassium phosphate salts were from Scharlau Chemicals (Barcelona, Spain). Orlistat was from Laboratorio Chile (Santiago, Chile). Culture medium, antibiotics and fetal bovine serum were obtained from Invitrogen Corp. (Waltham, MA, USA). Ultrapure water was obtained using a Barnsted EasyPure water filter (Thermo Scientific, Marietta, OH, USA).

### 3.2. Sample Preparation

Fruits of *G. decorticans* were collected in seven different locations of the Región de Atacama, Chile, namely, in the Provincia de Chañaral, Copiapó and Huasco (Figure 1C). From the Provincia de Chañaral: (1) Diego de Almagro (26°23′ S, 70°02′ W); and (2) Inca de Oro (26°45′ S, 69°54′ W); from the Provincia de Copiapó; (3) Copiapó (ripe and turning fruits) (27°40′ S, 70°13′ W); from the Provincia de Huasco; (4) road to Alto del Carmen (28°45′ S, 70°29′ W); (5) road to El Transito (28°52′ S; 70°17′ W) (two samples); (6) Pinte (28°58′ S, 70°17′ W); and (7) road to Conay (28°55′ S, 70°04′ W). All fruits were collected in February 2013, transported to the laboratory at room temperature and frozen until processing. The ripening process of chañar fruits occurs on the tree and is associated with water loss of the pulp. When the fruit is ripe, they fall down from the tree and are collected at this stage. The pulp is thick and with low water content. The fruits were air-dried and the edible portion (pulp) was mechanically separated from the husk and grinded. The edible pulp was separated from the husks and extracted with MeOH:H_2_O (7:3) under reflux for 15 min, two times. The dry fruit:solvent ratio was 1:3 *w*/*v*. The procedure was carried out in a flask fitted with a condenser, changing the solvent each time. The combined extracts of each sample were filtered, dried under reduced pressure and lyophilized. After freeze-drying, the extracts were dissolved in water, filtered and adsorbed into Amberlite XAD-7 resin. The resin was washed with water, and the phenolics were desorbed with MeOH to afford the PEE solution. This solution was dried under reduced pressure and lyophilized for analysis and bioactivity assays. Samples were stored refrigerated and in the dark to prevent oxidation.

### 3.3. Total Phenolic (TP), Total Flavonoid (TF) and Total Proanthocyanidin (TPAC) Content 

The TP was determined by the Folin-Ciocalteu method as previously described [7]. The results are expressed as g gallic acid equivalents (GAE)/kg PEE. The TF was determined by the AlCl_3_ method [7]. The results are expressed as g catechin equivalent (CE)/kg PEE. The TPAC was determined following the 4-dimethylaminocinnamaldehyde methodology [33] and the results are expressed as g CE/kg PEE.

### 3.4. Antioxidant Activity 

#### 3.4.1. Reducing Power

The ferric-reducing antioxidant power (FRAP) assay was carried out according to a previously described method [7]. The cupric-reducing antioxidant capacity (CUPRAC) assay was performed according to literature [34]. Results are expressed as mmoles Trolox equivalents/kg PEE. Catechin was used as a positive control in both assays.

#### 3.4.2. Scavenging of Free Radicals

The scavenging capacity of the *G. decorticans* PEEs was evaluated against the free radicals DPPH and superoxide anion. The discoloration of the DPPH radical was carried out according to literature [7]. The scavenging of the superoxide anion was evaluated following a previously described method [35]. Results are presented as the amount of extract that is able to scavenge 50% of the radicals (SC_50_, μg PEE/mL).

### 3.5. Inhibition of Pro-Inflammatory Enzymes 

#### 3.5.1. Lipooxygenase (LOX)

LOX inhibitory activity was determined following a previously reported methodology [36]. The assay mixture containing soybean lipoxygenase (166.6 U), sodium borate buffer (200 mM, pH 9.0), linoleic acid (134 μM) and chañar PEEs in DMSO (1–100 μg/mL). The assay mixture was pre-incubated at room temperature during 4 min, and absorbance was recorded every 30 s during 4 min at 234 nm. Results are expressed as IC_50_ (μg PEE/mL), corresponding to the concentration of the sample that inhibits the activity of LOX by 50%. Caffeic acid was used as a positive control.

#### 3.5.2. Cyclooxygenase (COX) 

COX inhibitory activity was determined using a commercial kit (560131, Cayman Chemical, Ann Arbor, MI, USA). Enzyme and samples (50 μg PEE/mL) were pre-incubated during 10 min and then arachidonic acid was added. The reaction was conducted for exactly 2 min at 37 °C and halted in boiling water for 5 min. The PGH_2_ produced was reduced to the more stable PGF_2α_ with stannous chloride and determined by enzyme immune assay (EIA) at 415 nm. Results were expressed as a percentage of inhibition of PGF_2α_ production. The commercial anti-inflammatory compound nimesulide was included as the reference compound [4].

#### 3.5.3. Secretory Phospholipase A2 (sPLA2)

The sPLA2 inhibitory activity was determined using a colorimetric kit (10004883, Cayman Chemical). Enzyme and samples (200–100 μg PEE/mL) were mixed, and then the substrate (diheptanoyl Thio-PC) was added, and incubated during 15 min at 25 °C. Then, DTNB (Ellman’s reagent) was added and absorbance was read at 415 nm. Results are expressed as a percentage of inhibition or IC_50_ (μg PEE/mL). Ursolic acid was used as the reference compound [37].

### 3.6. Inhibition of Metabolic Syndrome Associated Enzymes

#### 3.6.1. α-Glucosidase Inhibition Assay

The α-glucosidase inhibition assay was carried out as previously described [4]. Briefly, the reaction mixture contained sodium phosphate buffer (200 mM, pH 6.6), extract (10–0.1 μg PEE/mL), and α-glucosidase (0.25 U/L). After 15 min of pre-incubation at 37 °C, the reaction was started by adding *p*-nitrophenyl-α-d-glucopyranoside (5 mM) into the wells. The mixture was incubated for 15 min at 37 °C. Then, absorbance was measured at 415 nm in a microplate reader (ELx801, BioTek, Winooski, VT, USA). Results are expressed as IC_50_ values (μg PEE/mL). Acarbose was used as a positive control [4].

#### 3.6.2. α-Amylase Inhibition Assay

The α-amylase inhibition assay was carried out as described in literature [38]. Briefly, the PEEs (10–10 μg/mL) were co-incubated with 1% starch for 5 min at 37 °C, and then α-amylase solution (8 U/mL) was added and incubated for another 20 min. After the incubation, 400 µL of the color reagent (prepared mixing 20 mL 96 mM 3,5-dinitrosalicylic acid + 8 mL 5.31 M sodium potassium tartrate in 2 M NaOH + 12 mL distilled water) was added, mixed and incubated for 15 min in boiling water. Then, absorbance was measured in a microplate reader at 550 nm. Acarbose was included as the reference control [4]. Results are expressed as IC_50_ values (μg PEE/mL).

#### 3.6.3. Lipase Inhibition Assay

The assay was carried out using the *p*-nitrophenyl palmitate assay [39]. Porcine pancreatic lipase type II was re-suspended in ultrapure water at 20 mg/mL. The enzyme was centrifuged at 13,000 rpm at 4 °C for 10 min, and the supernatant was recovered for the assay. The substrate *p*-nitrophenyl palmitate (0.08% *w*/*v*) was prepared in 5 mM sodium acetate buffer (pH 5.0) containing 1% Triton X-100. This solution was heated in boiling water for 2 min for a better dissolution and cooled to room temperature. The assay mixture was 100 mM Tris buffer (pH 8.2), extracts, lipase and substrate solution. The mixture was incubated for 2 h at 37 °C and absorbance was read at 400 nm. All samples were assayed at 100 µg/mL as the maximum concentration in sextuplicate. Orlistat was used as the reference compound [4]. Results are expressed as IC_50_ values (μg PEE/mL) or as percentage of inhibition.

### 3.7. HPLC-DAD and HPLC-MS Analysis

The *G. decorticans* PEEs were analysed by HPLC-DAD to compare their phenolic profile. Taking into account the results of the bioactivity assays and the sample availability, the PEEs from Alto del Carmen and Copiapó were selected for the HPLC-MS^n^ studies. The HPLC system used was from Shimadzu (Shimadzu Corporation, Kyoto, Japan), and consisted of a LC-20AT pump, a SPD-M20A UV diode array detector, CTO-20AC column oven and LabSolution software. A MultoHigh 100 RP 18–5 µ (250 mm × 4.6 mm) (CS-Chromatographie Service GmbH, Langerwehe, Germany) column was used. The HPLC conditions included a linear gradient solvent system with 1.0 mL/min flow and 30 °C, consisting of two solvent systems: H_2_O–formic acid–acetonitrile (ACN) (87:5:3, *v*/*v*/*v*, system A) and H_2_O–formic acid–ACN (40:5:50, *v*/*v*/*v*, system B). Initial conditions were 97% A and 3% B. Then, the solvent ratio was changed to 63:27 A:B in 100 min and returned to the initial conditions (97:3 A:B) at min 105. The column was allowed to stabilize for additional 10 min in the same gradient (95:5 A:B) for the next injection. The UV spectra from the chromatograms were recorded from 200 to 600 nm for peak characterization. 

The HPLC-ESI-MS/MS analyses were recorded using an Agilent 1100 liquid chromatography system (Agilent Technologies Inc., Santa Clara, CA, USA) connected through a split to an Esquire 4000 Ion Trap LC/MS^n^ system (Bruker Daltoniks, Bremen, Germany). Ionization was performed at 3000 V assisted by nitrogen as nebulizing gas at 50 psi and as drying gas at 365 °C, with a flow rate of 10 L/min. Negative ions were detected using full scan (*m*/*z* 2000–20) and normal resolution (scan speed 10,300 *m*/*z*/s; peak with 0.6 FWHM/*m*/*z*). The trap parameters were set in ion change control (ICC) using manufacturer default parameters, and maximum accumulation time of 200 ms. The mass spectrometric conditions for analysis were: electrospray needle, 4000 V; end plate offset, −500 V; skimmer 1, 56.0 V; skimmer 2, 6.0 V; capillary exit offset, 84.6 V; capillary exit, 140.6 V. Collision induced dissociation (CID) spectra were obtained with a fragmentation amplitude of 1.00 V (MS/MS) using helium as the collision gas and was automatically controlled through the SmartFrag option. The same column and solvent system used in HPLC-DAD chromatography were used and the compounds were monitored at 280 nm.

### 3.8. Fractionation of PEEs in Sephadex LH-20

In order to get a more comprehensive analysis of the polyphenol composition of *G. decorticans*, a Sephadex column chromatography was applied. For this purpose, the PEE from Diego de Almagro (2.5 g) was dissolved in MeOH:H_2_O 7:3 (*v*/*v*) and was permeated on a Sephadex LH-20 column (column length: 112 cm, internal diameter 5.1 cm, filled with Sephadex LH-20 to 48 cm) using MeOH:H_2_O 7:3 as the eluent. Some 48 fractions of 20–40 mL each were collected. TLC analysis was carried out in silica gel plates (Sigma-Aldrich, Steinheim, Germany) using as the mobile phase the mixture ethyl acetate:acetic acid:H_2_O (10:2:3 *v*/*v*/*v*), revealed with diphenylboric acid-ethanolamine complex in MeOH and visualized under UV/visible light. Fractions with similar TLC patterns were pooled into twelve sub-fractions as follows: 1–9; 10–14; 15–19; 20–25; 26–27; 28–30; 31–33; 34–37; 38–42; 43–44; 45–46 and 47–48. Fractions were submitted to the DPPH and α-glucosidase assays, as well as HPLC-DAD and HPLC-MS analyses.

### 3.9. Cytotoxicity Assay 

Human lung fibroblast MRC-5 (ATCC CCL-171) were grown as monolayers in minimum essential Eagle medium (MEM) with Earle’s salts and supplemented with 2 mM l-glutamine, 1.5 g/L sodium bicarbonate, 10% heat-inactivated fetal bovine serum (FBS), 100 IU/mL penicillin and 100 µg/mL streptomycin. Cells were cultured in a humidified incubator with 5% CO_2_ in air at 37 °C. For the subsequent experiments, cells were plated at a density of 2.5 × 10^4^ cells/mL.

Confluent cultures of MRC-5 cells were treated during 24 h with medium containing the different PEEs at concentrations ranging from 0–500 µg/mL. The samples were dissolved in medium supplemented with 2% FBS. Cells treated with medium only were used as controls. Cell viability was determined at the end of the incubation by means of the MTT reduction assay [40]. Results were transformed to percentage of 100% viability control and were calculated from the dose-response curves.

### 3.10. Statistical Analyses 

All experiments were carried out in triplicate. All data are presented as mean values ± standard deviation (SD). Statistical significant differences within each sample were determined by one-way analysis of variance (ANOVA) followed by Tukey’s multiple comparison test (*p <* 0.05). To assess the relationship between the bioactivities and composition, Pearson’s correlation coefficients were calculated with 95% confidence, using the SPSS 14.0 software for Windows (IBM, Armonk, NY, USA).

## 4. Conclusions

The constituents of the Chilean *G. decorticans* fruit PEE include (epi)-catechin derivatives, procyanidin dimer, trimer and tetramers, ellagic acid derivatives and flavonoids glycosides. The total proanthocyanidin content of the Chilean *G. decorticans* pulp was positively correlated with the antioxidant activity and the inhibition of the enzyme α-glucosidase. The compounds tentatively identified in the active antioxidant fractions from the whole PEE of Chilean chañar includes procyanidin B-type dimer, procyanidin A trimer, glycosides of kaempferol, isorhamnetin and quercetin, (epi)-catechin hexosides, as well as dicaffeoylquinic acids. The high inhibitory activity of the PEE on the enzyme α-glucosidase and the fact that chromatographic separation of the whole extract yielded fraction pools with even higher inhibitory effect on the enzyme than the whole PEE encourages further work to isolate and identify the active α-glucosidase inhibitors from the fruit. Our findings support a different chemistry for the western Andean population of *G. decorticans*. Additional research is needed to study the genetic similarities between both populations of this native food plant.

## Figures and Tables

**Table 1:** Pulp percent, yields of methanol extract (MeOH) and phenolic-enriched extract (PEE), total phenolic (TP), total flavonoid (TF), total proanthocyanidin (TPAC) content and antioxidant activity of *G. decorticans* fruit PEEs.

Collection Place	Pulp (%)	MeOH (%)	PEE (%)	TP (g GAE/kg PEE)	TF (g CE/kg PEE)	TPAC (g CE/kg PEE)	FRAP (mmol TE/g PEE)	CUPRAC (mmol TE/g PEE)	DPPH SC_50_ (μg/mL)	O_2_ Scavenging SC_50_ (μg/mL) ^#^
*Provincia de Chañaral*										
Diego de Almagro	78.1	43.6	1.7	389.1 ± 2.0 ^a^	222.1 ± 1.7 ^a^	24.5 ± 2.7 ^a^	1.8 ± 0.1 ^a^	4.1 ± 0.1 ^a^	12.1 ± 0.1 ^a^	36.6 ± 0.9 ^a^
Inca de Oro	58.3	29.1	0.8	639.2 ± 11.1 ^b^	260.5 ± 8.9 ^b^	84.1 ± 7.4 ^b^	3.1 ± 0.1 ^b^	6.7 ± 0.3 ^b^	5.3 ± 0.7 ^b^	18.1 ± 1.0 ^b^
*Provincia de Copiapó*										
Copiapó (turning)	66.6	51.7	1.8	450.0 ± 4.6 ^c^	55.8 ± 2.1 ^c,d^	90.1 ± 4.7 ^b^	2.9 ± 0.1 ^b^	7.4 ± 0.1 ^c^	4.9 ± 0.9 ^b^	15.2 ± 1.2 ^b,c^
Copiapó (ripe)	67.4	52.7	1.6	446.3 ± 2.8 ^c^	50.3 ± 2.4 ^c^	90.2 ± 4.1 ^b^	2.8 ± 0.2 ^b^	6.3 ± 0.1 ^b^	3.9 ± 0.5 ^b^	30.1 ± 0.9 ^d^
*Provincia de Huasco*										
Alto del Carmen	59.0	43.9	4.0	508.3 ± 4.1 ^d^	37.8 ± 1.6 ^e^	123.4 ± 1.1 ^c^	3.0 ± 0.2 ^b^	6.7 ± 0.1 ^b^	3.9 ± 0.7 ^b^	14.5 ± 0.3 ^c^
El Transito (1)	73.2	45.9	1.2	236.4 ± 2.2 ^e^	163.5 ± 1.4 ^f^	11.7 ± 1.8 ^d^	1.6 ± 0.1 ^a,c^	3.4 ± 0.1 ^d^	10.4 ± 0.5 ^a^	44.2 ± 0.4 ^e^
El Transito (2)	67.7	53.8	0.9	361.0 ± 0.0 ^f^	77.1 ± 2.5 ^g^	17.7 ± 3.4 ^a,d^	1.4 ± 0.1 ^c^	1.3 ± 0.1 ^e^	19.3 ± 0.9 ^c^	45.1% ± 3.5 ^#^
Pinte	74.3	69.2	2.0	369.1 ± 0.0 ^f^	65.1 ± 2.6 ^d,h^	48.3 ± 1.2 ^e^	1.9 ± 0.1 ^a^	5.5 ± 0.2 ^f^	5.2 ± 0.6 ^b^	32.5 ± 2.1 ^d^
Conay	77.0	59.4	0.7	196.2 ± 1.5 ^g^	116.0 ± 3.0 ^i^	BDL	0.9 ± 0.1 ^d^	2.5 ± 0.1 ^g^	24.3 ± 0.3 ^d^	n.d.
Catechin *							5.4 ± 0.1	13.4 ± 0.3	11.4 ± 1.6	8.7 ± 0.1

* Reference compound; BDL: below detection limits. ^#^ % of inhibition. Different letters (^a–i^) in the same column show significant differences with each sample, according to Tukey’s test (*p* < 0.05).

**Table 2:** Inhibition of the pro-inflammatory enzymes (LOX, COX-1/COX-2 and sPLA_2_) and metabolic syndrome-associated enzymes (α-glucosidase and lipase) by *G. decorticans* fruit PEEs. Data are reported as IC_50_ values or % inhibition (at 50 μg/mL for COX-1/ COX-2, 200 μg/mL for PLA_2_ and 100 μg/mL for lipase).

Collection Place	LOX IC_50_ (μg PEE/mL)	COX-1% Inhibition	COX-2% Inhibition	sPLA_2_% Inhibition or IC_50_ (μg PEE/mL)	α-Glucosidase IC_50_ (µg PEE/mL)	Lipase% of Inhibition or IC_50_ (µg PEE/mL)
*Provincia de Chañaral*						
Diego de Almagro	60.1 ± 3.2 ^a,b^	61.8 ± 1.3 ^a^	60.5 ± 1.2 ^a^	98.9 ± 0.8 ^a^	4.7 ± 0.0 ^a^	17.1 ± 0.9 ^#^
Inca de Oro	61.2 ± 4.4 ^a,b^	80.9 ± 1.2 ^b^	18.9 ± 1.2 ^b^	142.9 ± 4.9 ^b^	4.5 ± 0.3 ^a^	9.9 ± 0.7 ^a^
*Provincia de Copiapó*						
Copiapó (turning)	51.8 ± 1.6 ^a,c^	74.8 ± 1.5 ^c^	12.9 ± 0.2 ^c^	38.1 ± 1.9 ^#^	2.1 ± 0.1 ^b^	66.0 ± 1.9 ^b^
Copiapó (ripe)	53.1 ± 1.7 ^a,b^	92.1 ± 1.3 ^d^	55.5 ± 0.6 ^d^	42.8 ± 1.5 ^#^	0.8 ± 0.0 ^c^	29.7 ± 3.0 ^#^
*Provincia de Huasco*						
Alto del Carmen	43.6 ± 3.2 ^c^	Inactive	25.9 ± 0.6 ^e^	156.0 ± 1.2 ^c^	0.7 ± 0.1 ^c^	14.2 ± 0.1 ^c^
El Transito (1)	74.0 ± 3.6 ^d^	Inactive	51.3 ± 0.4 ^f^	39.9 ± 0.1 ^#^	5.0 ± 0.3 ^a^	33.6 ± 3.4 ^#^
El Transito (2)	>100	70.1 ± 1.2 ^e^	31.2 ± 0.6 ^g^	34.7 ± 0.1 ^#^	7.3 ± 0.4 ^d^	34.9 ± 3.5 ^#^
Pinte	96.8 ± 1.8 ^e^	87.3 ± 1.3 ^f^	Inactive	34.6 ± 0.3 ^#^	4.9 ± 0.1 ^a^	0.0 ^#^
Conay	76.8 ± 2.8 ^d,e^	80.8 ± 1.1 ^d,f^	76.0 ± 0.9 ^h^	n.d.	n.d.	n.d.
Caffeic acid *	37.2 ± 2.0					
Nimesulide *		100	100			
Ursolic acid *				26.7 ± 0.7^#^		
Acarbose *					120.9 ± 2.0	
Orlistat *						0.04 ± 0.00

* Reference compounds; n.d.: not determined. ^#^ % of inhibition. Different letters (^a–h^) in the same column show significant differences with each sample, according to Tukey’s test (*p* < 0.05).

**Table 3:** Tentative identification of compounds in the phenolic-enriched extract of *G. decorticans* fruits by HPLC-DAD-MS.

Peak	*R*_t_ (min)	λ Max (nm)	[M − H]^−^ (*m*/*z*)	MS/MS (*m*/*z*)	Tentative Identification
**1**	22.1	307sh, 280	463	301	Ellagic acid hexoside 1
**2**	26.0	280	433	301	Ellagic acid pentoside 1
**3**	27.2	325, 298sh, 280	421	287, 151	Eriodictyol pentoside
**4**	34.7	280	451	289	(epi)-catechin hexoside 1
**5**	41.1	280	451	289	(epi)-catechin hexoside 2
**6**	43.9	274	577	559, 451, 425, 407, 289	Procyanidin B-type dimer 1 [27]
**7**	46.1	327sh, 280	465	303	Taxifolin hexoside 1
**8**	46.3	280	451	289	(epi)-catechin hexoside 3
**9**	47.6	283	329	167, 123	Vanillic acid hexoside
**10**	50.2	324sh, 298sh, 280	461	419, 401, 341, 299, 209, 167	Vanillic acid hexoside pentoside
**11**	52.0	ND	615	493, 405, 327, 285	Kaempferol derivative
**12**	56.0	279	577	471, 451, 425, 289	Procyanidin B-type dimer 2 [27]
**13**	56.1	279	863	711, 573, 451, 289	Procyanidin A-type trimer 1 [28]
**14**	57.4	327sh, 280	465	303	Taxifolin hexoside 2
**15**	59.8	306sh, 280	449	287, 151	Eriodictyol hexoside
**16**	61.6	274	863	711, 695, 573, 451	Procyanidin A-type trimer 2 [28]
**17**	64.1	279	863	711, 573, 451	Procyanidin A-type trimer 3 [28]
**18**	64.4	339, 273	337	191	5-*p*-coumaroylquinic acid [29]
**19**	66.2	279	1153	863, 575, 451, 289	Procyanidin B-type tetramer [27]
**20**	69.1	347, 280	771	609, 301	Quercetin dihexoside rhamnoside 1
**21**	71.3	279	939	863, 573, 411, 289	Procyanidin trimer derivative
**22**	72.6	347, 280	741	579, 447, 285	Kaempferol dihexoside pentoside
**23**	73.3	343, 280	625	463, 301	Quercetin dihexoside 2
**24**	73.5	350, 280	771	609, 463, 301	Quercetin dihexoside rhamnoside
**25**	73.8	373, 250	479	317	Myricetin hexoside
**26**	75.4	279	577	539, 449, 289	Procyanidin B-type dimer 3 [28]
**27**	75.6	347, 277	755	593, 575, 285	Kaemperol hexoside rutinoside
**28**	76.2	317sh, 280	565	433, 271	Naringenin hexoside pentoside
**29**	77.5	279	577	539, 423, 289	Procyanidin B-type dimer 4 [28]
**30**	78.2	317sh, 280	433	271	Naringenin hexoside 1
**31**	78.4	316sh, 280	623	301	Ellagic acid derivative
**32**	79.1	355, 317sh, 280	595	301	Quercetin pentoside hexoside 1
**33**	80.3	355, 255	771	609, 463, 301	Quercetin dihexoside rhamnoside 2
**34**	80.5	365, 265	609	447, 429, 285	Kaempferol dihexoside
**35**	80.8	365, 280	623	285	Kaempferol hexoside glucuronide
**36**	81.6	365, 300sh, 260	609	429, 285	Kaempferol dihexoside 2
**37**	81.6	300sh, 288	433	271	Naringenin hexoside 2
**38**	84.1	ND	579	447, 285	Kaempferol pentoside hexoside
**39**	84.3	355, 255	609	463, 301	Rutin ^a^
**40**	85.8	340, 267, 254	755	285, 241, 175	Luteolin rhamnoside dihexoside
**41**	86.5	ND	463	301	Quercetin hexoside
**42**	87.0	342, 268, 250	609	477, 315	Isorhamnetin pentoside hexoside
**43**	87.6	355, 255	477	301	Quercetin glucuronide
**44**	88.6	ND	579	447, 285	Kaempferol hexoside pentoside 2
**45**	88.7	342, 268, 250	755	623, 315	Isorhamnetin rutinoside pentoside
**46**	89.0	342, 268, 250	609	477, 315	Isorhamnetin pentoside hexoside 2
**47**	90.8	350, 268, 247	785	623, 315	Isorhamnetin dihexoside rhamnoside
**48**	93.4	320, 280	515	353, 191, 135	3,5-dicaffeoylquinic acid [29]
**49**	93.1	343, 268	609	477, 315, 301	Isorhamnetin pentoside hexoside 3
**50**	94.6	343, 270	623	315	Isorhamnetin rutinoside
**51**	96.9	340, 300sh, 280	447	285	Kaempferol hexoside
**52**	97.5	ND	461	285	Kaempferol glucuronide
**53**	98.4	330, 245	515	353, 173	4,5-dicaffeoylquinic acid [29]

^a^ Identity confirmed by co-injection of authentic reference standard. All other assignments are tentative. ND: not detected.

**Table 4:** Percent distribution of constituents in the whole PEE of *G. decorticans* fruits from Diego de Almagro after gel permeation (Sephadex LH-20), DPPH and α-glucosidase inhibition by the different fractions. The antioxidant effect on the DPPH discoloration assay is shown as percent inhibition at 100 µg/mL or as SC_50_ values (µg/mL). The inhibitory activity towards α-glucosidase is presented as IC_50_ values (µg/mL).

Sample	Mass (mg)	% Fraction	DPPH (SC_50_, µg/mL)	α-Glucosidase (IC_50_, µg/mL)
**PEE**	2520	100	12.1 ± 0.1	4.7 ± 0.1
**Fractions**				
1–9	68.9	4.4	15.0 ± 0.0 ^#^	8.0 ± 0.4
10–14	217.1	14.0	16.1 ± 0.0 ^#^	7.3 ± 0.1
15–19	659.3	42.6	87.1 ± 0.1	2.6 ± 0.1
20–25	183.3	11.8	37.8 ± 0.0	0.7 ± 0.1
26–27	43.1	2.8	32.9 ± 0.0	0.6 ± 0.0
28–30	97.8	6.3	28.4 ± 0.0	0.7 ± 0.1
31–33	58.1	3.8	29.2 ± 0.0	0.6 ± 0.0
34–37	78.1	5.0	27.7 ± 0.0	0.4 ± 0.0
38–42	57.4	3.7	24.5 ± 0.0	0.5 ± 0.0
43–44	5.6	0.4	24.1 ± 0.0	0.9 ± 0.0
45–46	21.5	1.4	28.8 ± 0.1	0.6 ± 0.0
47–48	58.8	3.8	40.7 ± 0.1	0.5 ± 0.0
Acarbose *				120.9 ± 2.0
Catechin *			11.4 ± 1.6	

* Reference compounds; ^#^ % of inhibition.
